# Supplementary material for: Neural processing of goal and non-goal-directed movements on the smartphone
Source: Neuroimage Rep. 2023 Mar 15;3(2):100164. doi: 10.1016/j.ynirp.2023.100164 (PMC12172746; doi:10.1016/j.ynirp.2023.100164)
Supplement: Supplementary Methods [file mmc1.pdf]

Supplementary methods

Supplementary Methods Figure 1. Example movement sensor signals.......... 1

Supplementary Methods Figure 2. Participant selection diagram.......... 2

Supplementary Methods Figure 3. Alignment model methods and results.......... 3

Supplementary Methods Table 1. Alignment model hyperparameters.......... 4

Supplementary Methods Figure 4. Alignment model correction.......... 5

Supplementary Methods Table 2. Goal and non-goal-directed movement identification model hyperparameters.......... 6

Supplementary Methods Figure 5. Participant selection based on kinematic profile.......... 7

Supplementary Methods Figure 6. Goal and non-goal-directed movements during smartphone apps for selected participants.......... 8

Supplementary Methods Figure 7. Goal and non-goal-directed movements during smartphone apps for removed participants.......... 8

Supplementary Methods Figure 8. Number of goal and non-goal-directed trials before and after artifact rejection (bandpass filtered 1 to 30).......... 9

Supplementary Methods Figure 9. Number of goal and non-goal-directed trials before and after artifact rejection (bandpass filtered 1 to 45).......... 9

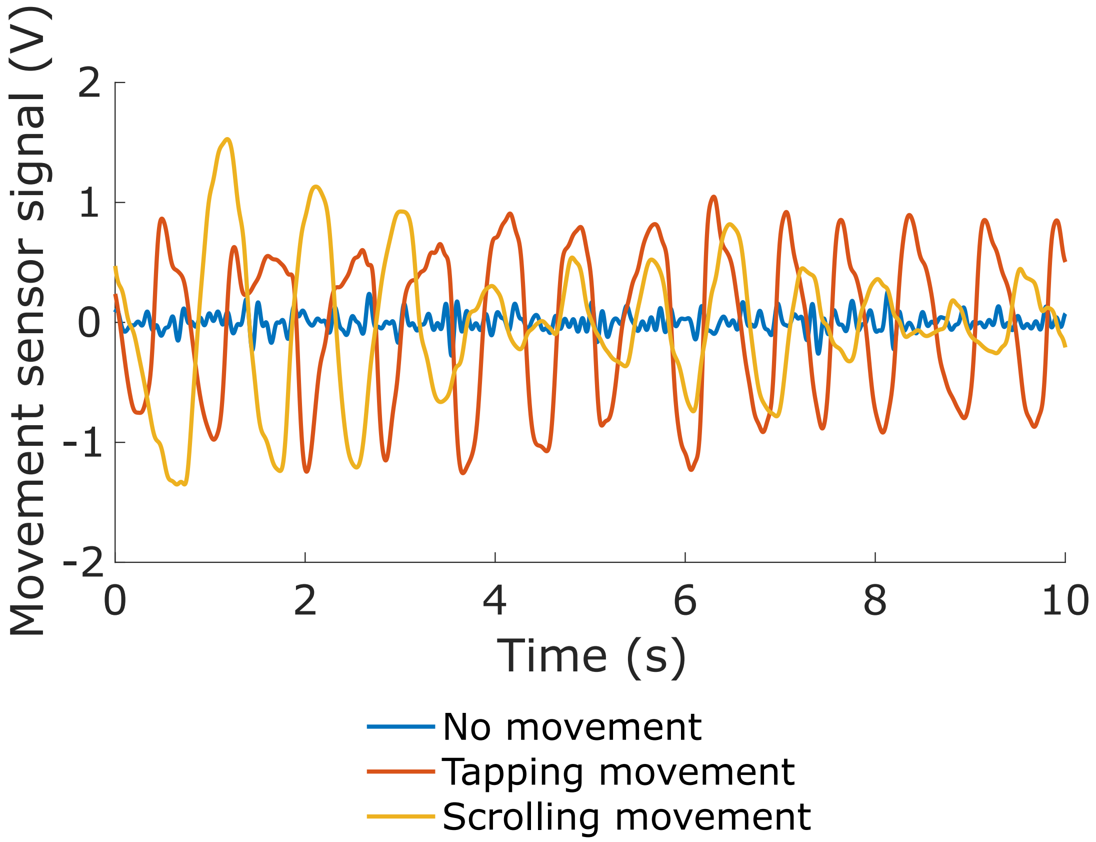

Supplementary Methods Figure 1. Common movements generated during smartphone use compared to no movements as measurement by the movement sensor.

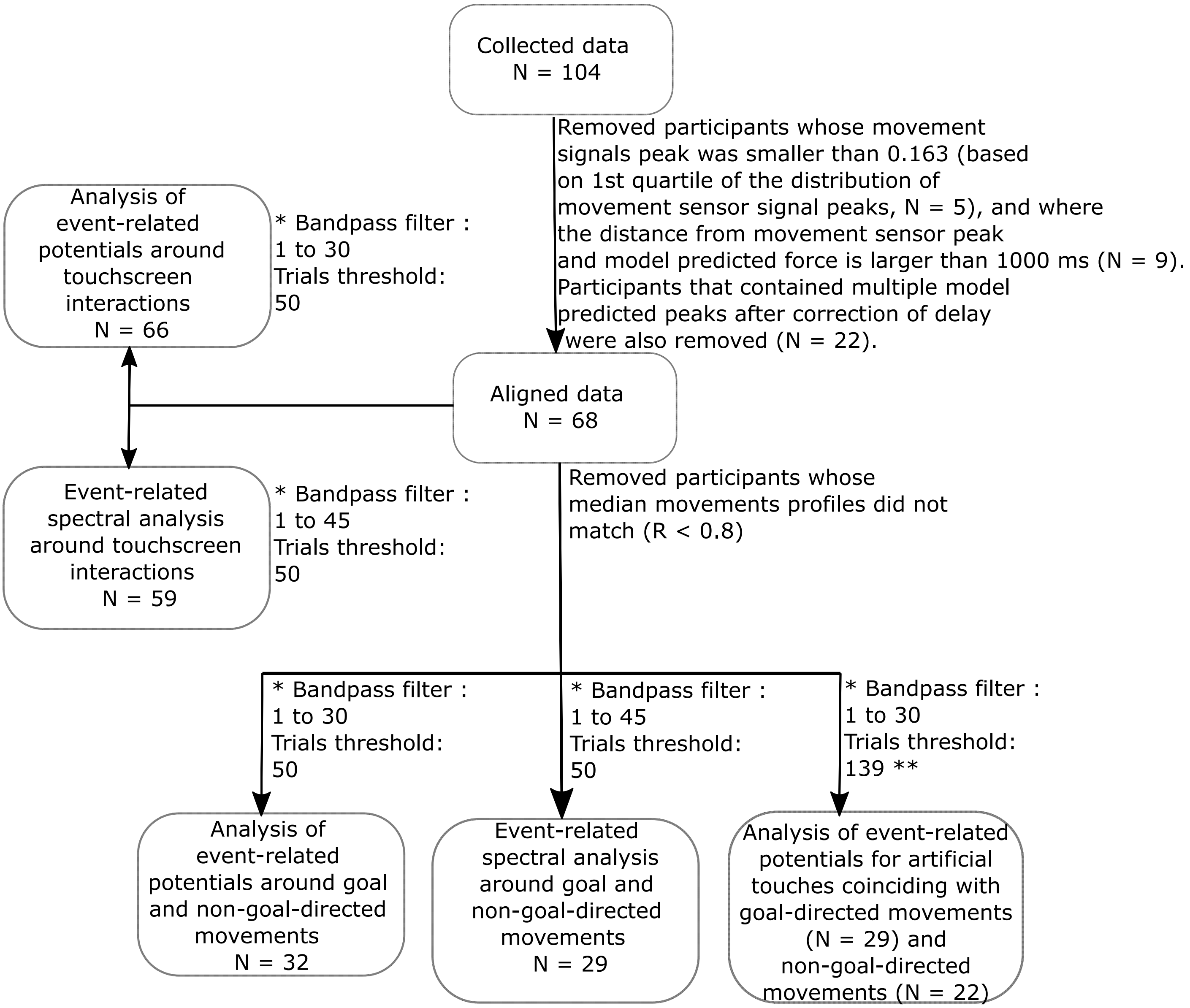

Supplementary Methods Figure 2. Participant selection at multiple stages of analysis.

* Participants were removed through the combination of a bandpass filter, artifact rejection (+/- 80 μV) and not enough trials for each event remaining.

** Threshold based on the minimum number of goal-directed movements encountered.

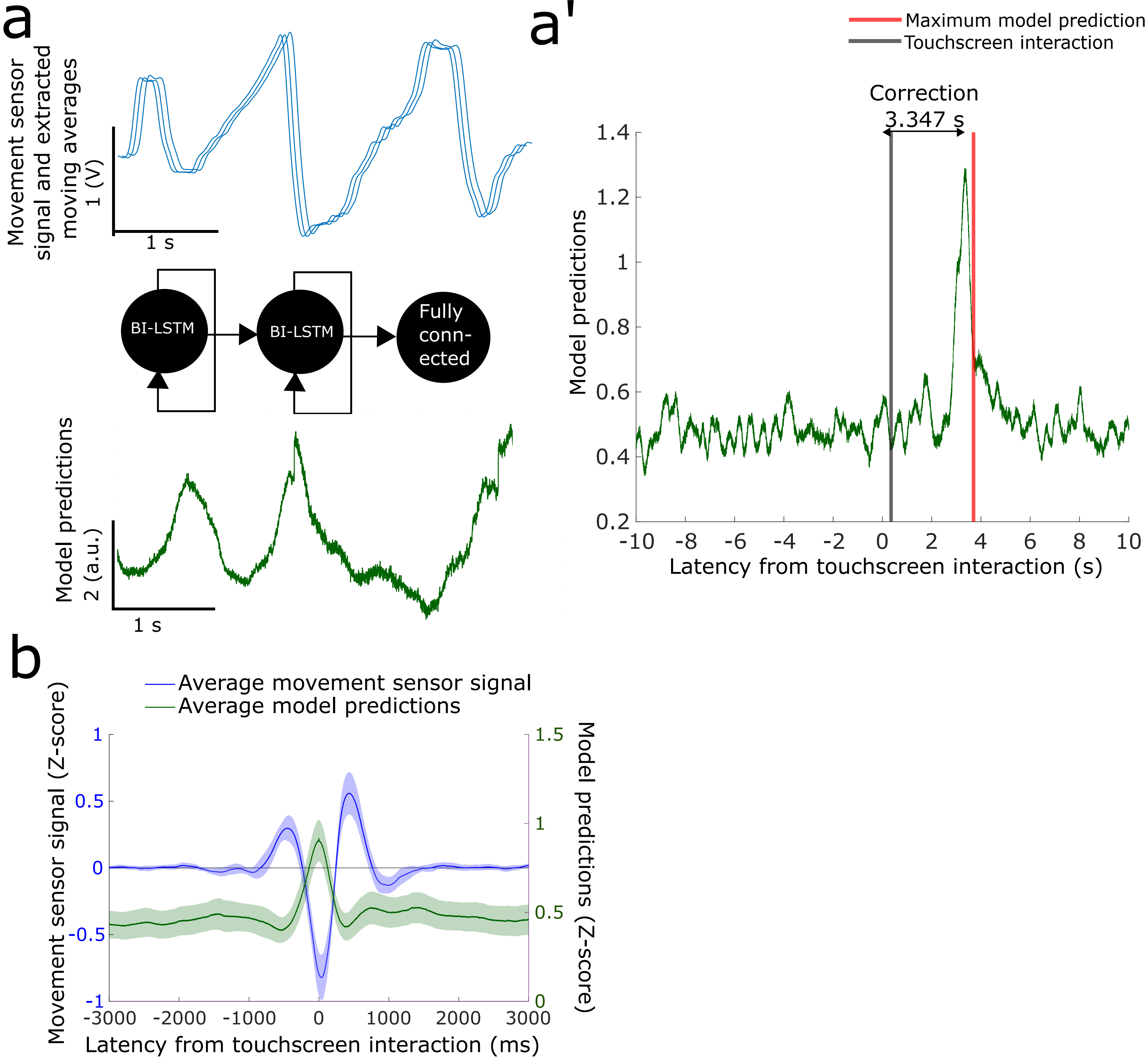

Supplementary Methods Figure 3. Alignment of smartphone data to EEG and movement sensor signals. **(a)** A BI-LSTM model was trained with movement sensor signals and 100 extracted moving averages to predict force sensor values. **(a')** The alignment was performed by correcting for the delay between the average predicted signal and the recorded touchscreen interaction. **(b)** Grand average of the movement sensor signals after alignment indicate that the lowest point of the bend of the thumb is near the touchscreen interaction (Z-score normalized for visualization). Grand average model predictions after alignment show that the model identified the location of the touchscreen interactions (Z-score normalized for visualization). Overall indicating successful alignment of the data.

Supplementary Methods Table 1

Hyperparameters for alignment model

Parameter	Value
Train/Validation/Test split	80/10/10
Total number of trainable parameters	425.480
Optimizer	adam
Learning rate	0.001
Loss	Mean squared error
Max epoch	5

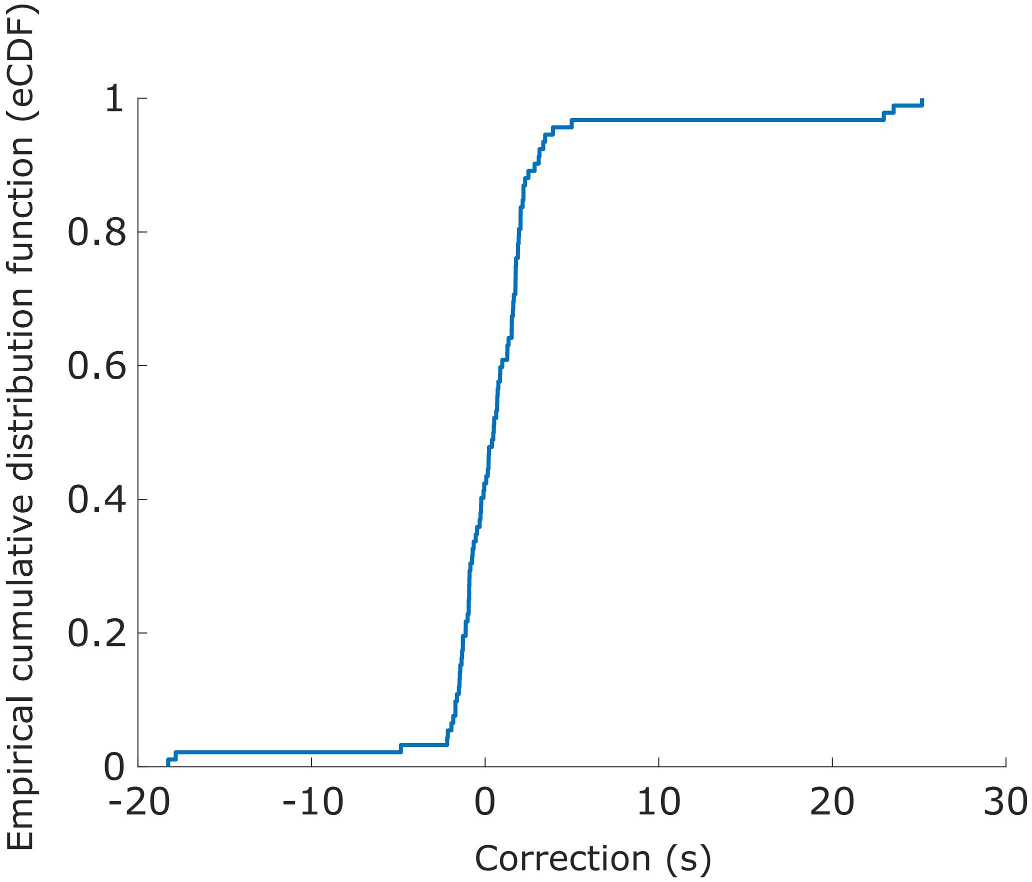

Supplementary Methods Figure 4. Alignment model correction. For each participant, the smartphone data and the movement data were aligned by correcting for the delay between the model predicted force and the touchscreen interaction.

Supplementary Methods Table 2

Hyperparameters for artificial neural networks trained to identify goal and non-goal-directed movements

Parameter	Value
Train/Validation/Test split	80/10/10
Total number of trainable parameters	66.689
Optimizer	RMSprop
Starting learning rate	0.0001
Loss	Binary Cross Entropy
Max epoch	100

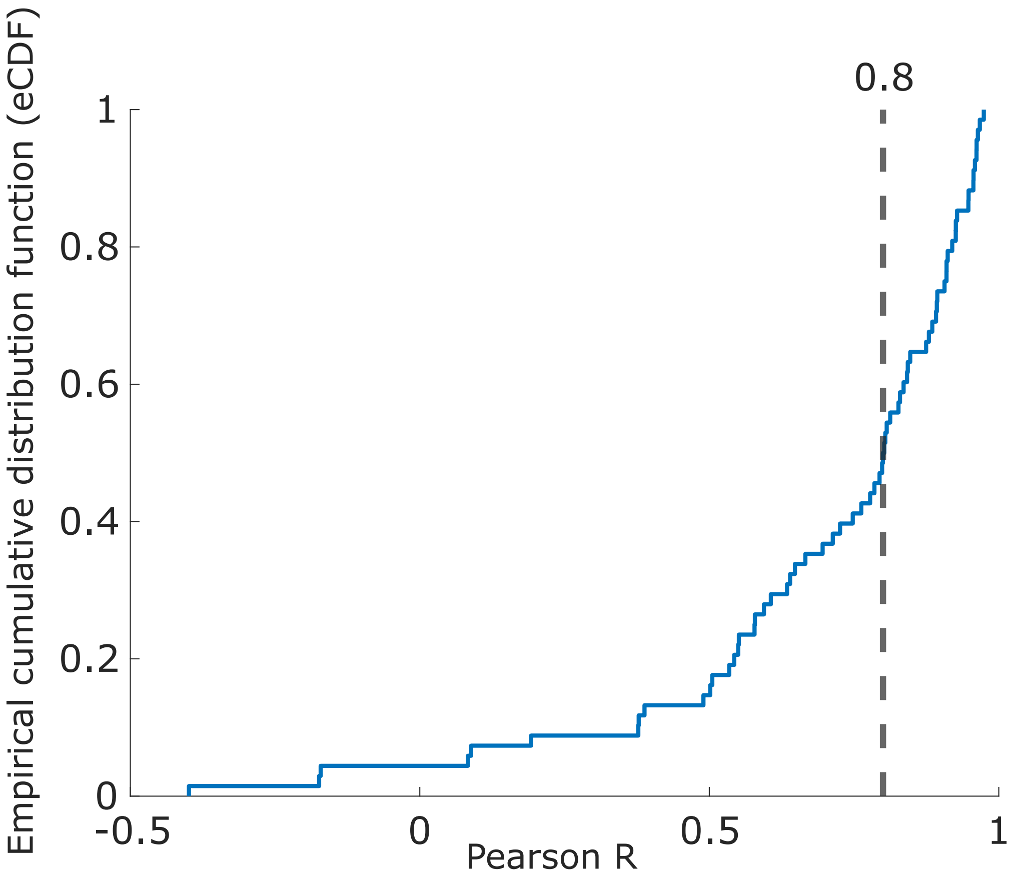

Supplementary Methods Figure 5. Participants with similar kinematic profiles were selected for EEG analysis based on a Pearson R larger than 0.8 (calculated over median z-normalized goal and non-goal-directed movements).

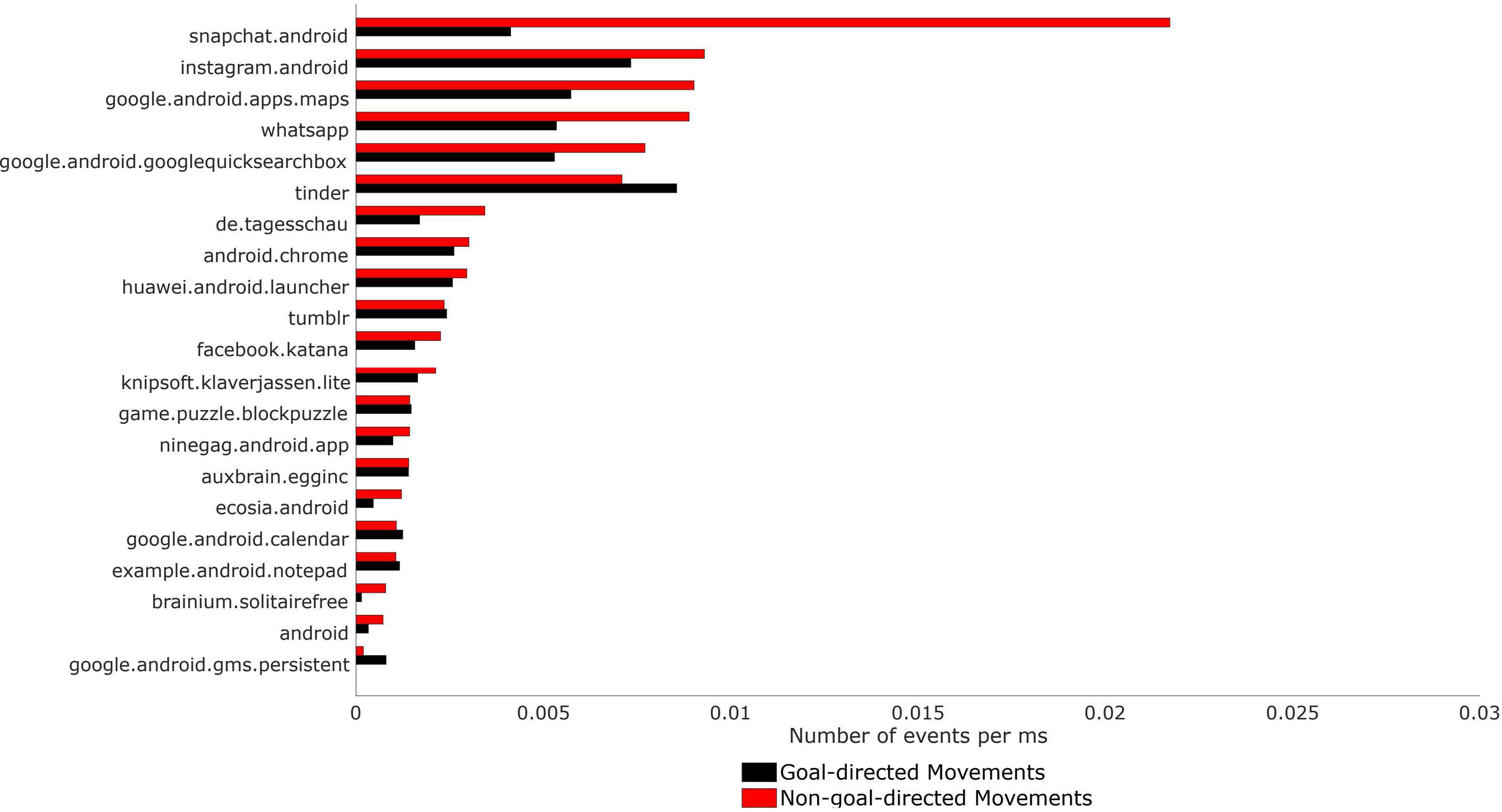

Supplementary Methods Figure 6. Frequency of goal-directed and non-goal-directed movements during use of smartphone apps for selected participants (N=32)

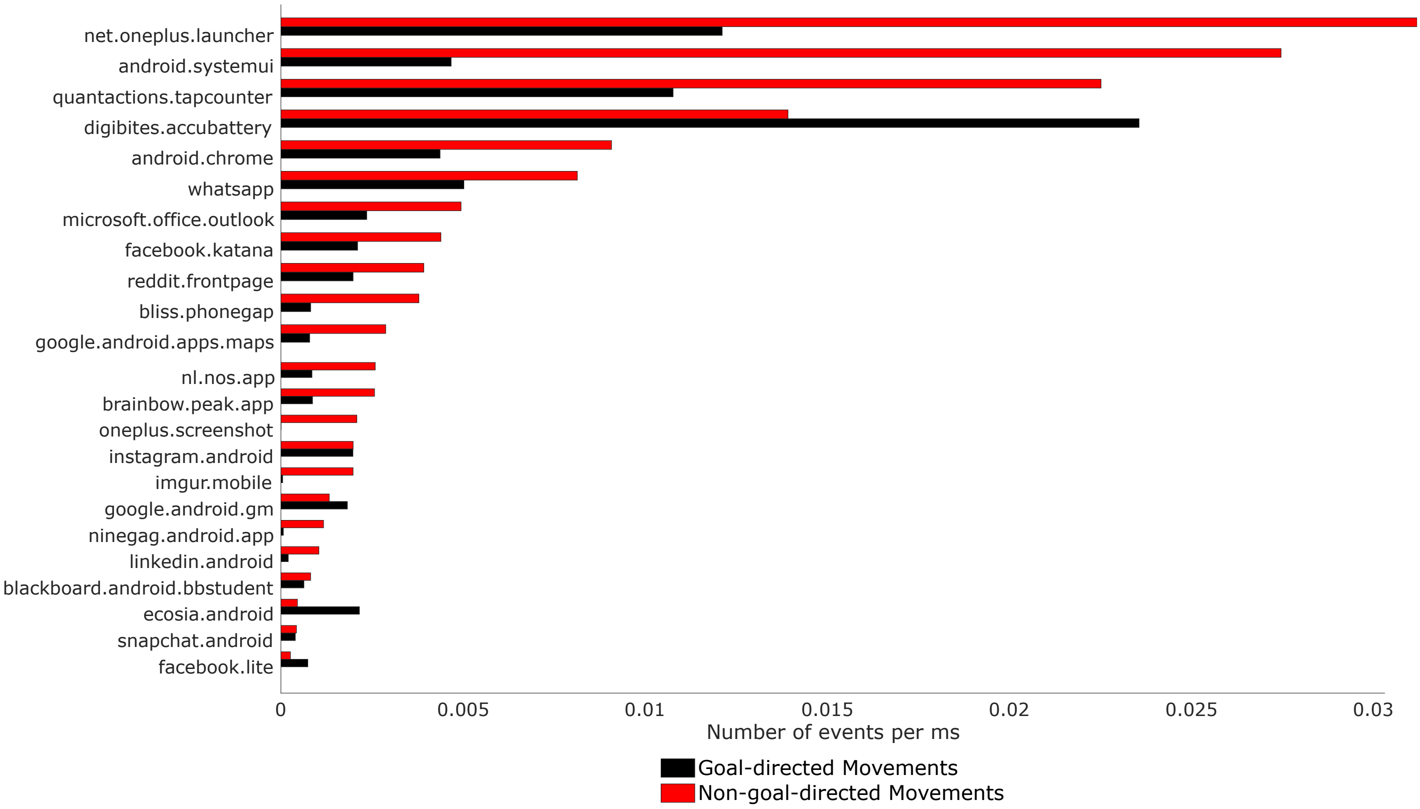

Supplementary Methods Figure 7. Frequency of goal-directed and non-goal-directed movements during use of smartphone apps for participants removed through dissimilar movement profiles (N=36)

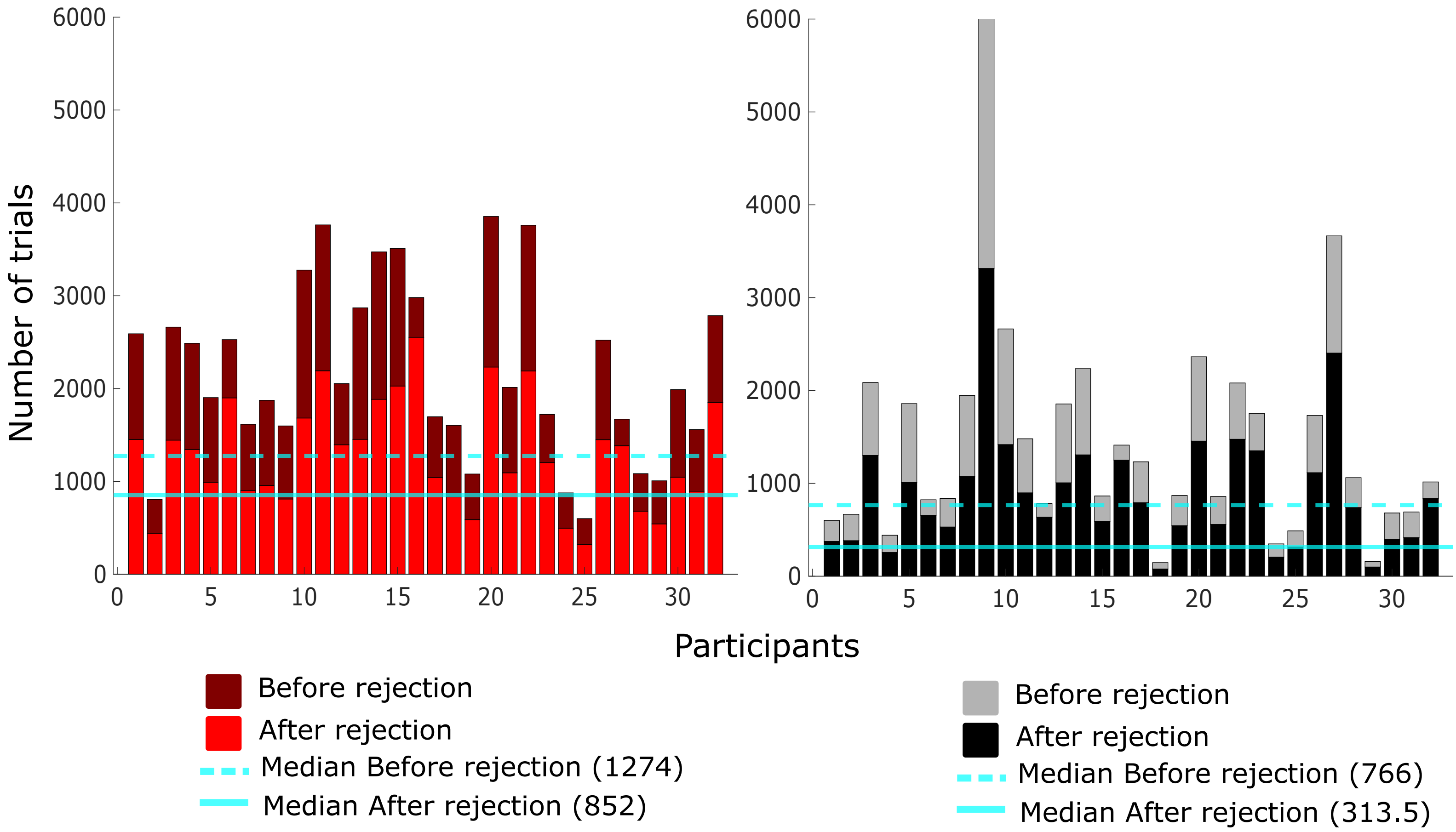

Supplementary Methods Figure 8. Number of trials of goal-directed and non-goal-directed movements before and after artifact rejection (bandpass filtered 1 to 30)

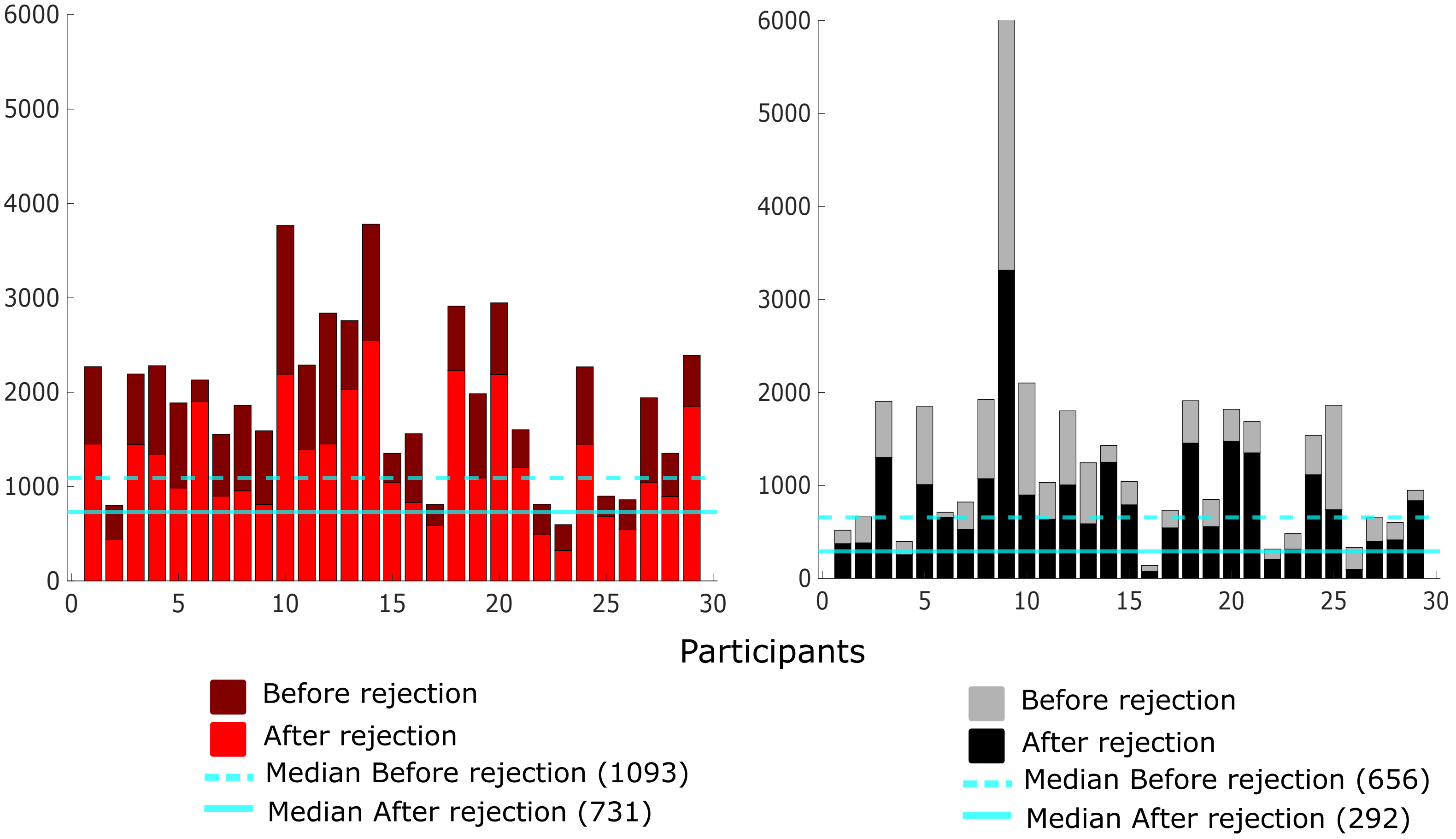

Supplementary Methods Figure 9. Number of trials of goal-directed and non-goal-directed movements before and after artifact rejection (bandpass filtered 1 to 45)
