# Supplementary material for: Neural processing of goal and non-goal-directed movements on the smartphone
Source: Neuroimage Rep. 2023 Mar 15;3(2):100164. doi: 10.1016/j.ynirp.2023.100164 (PMC12172746; doi:10.1016/j.ynirp.2023.100164)
Supplement: Supplementary Legends [file mmc2.pdf]

Neural processing of goal and non-goal-directed movements on the smartphone

Ruchella Kock, Enea Ceolini, Lysanne Groenewegen, and Arko Ghosh

Supplementary Legends

Supplementary Figure 1. Identification of goal and non-goal-directed movements. **(a)** An artificial neural network was trained to identify touchscreen interactions based on movement sensor signals. The movement signal approximate integrals over 1 ms windows were used as input. **(a')** The model well-identified movements surrounding the touchscreen interactions (goal-directed movements), but also identified highly similar movements which did not result in a touchscreen interaction (non-goal-directed movements). **(b)** The probability density of the number of events per ms for both goal and non-goal-directed movements (log_{10} normalized) shows that the two movement types occurred at a similar rate (N = 32). **(c)** The probability density of inter-event intervals for goal and non-goal-directed movements shows a relatively broad distribution for the latter (log_{10} normalized). Overlayed is the median across all participants. **(d)** Probability density of distance to non-goal-directed movements (log_{10} normalized). Non-goal-directed movements were more likely to occur after a goal-directed movement. The median distances across the participants are overlayed.

Supplementary Figure 2. Movement signal traces for goal (red, left) and non-goal-directed (black, right) movements time-locked to the predicted events for all participants (Z-score normalized for visualization). Overlaid are the median movement signals. The similarity between the movement types is indicated by the Pearson R.

Supplementary Figure 3. Grand averages of artificial neural network predictions (larger than optimal F2 score threshold denoted by dashed line) and probability distribution of the model predicted peaks in the sampled population (N = 32).

Supplementary figure 4. Scalp topologies of event-related changes in the alpha-band (8 to 11 Hz) for **(a)** goal- directed and **(b)** non-goal-directed movements. Significant statistical clusters were identified using one-sample *t*-tests and multiple comparisons corrected (MCC, $p < 0.05$, N = 29).

Supplementary figure 5. P-values of the statistical analysis for event-related (spectral) potentials of the goal and non-goal-directed movements.

Supplementary figure 6. P-values of the statistical analysis for event-related (spectral) potentials of the goal and non-goal-directed movements. **(a-a')** P-values for the movements coinciding with an artificial touch. **(b-b')** P-values for the movements for event-related spectral potentials collapsed over the alpha-band.

Supplementary Table 1. Descriptive statistics for artificial neural networks trained to identify the goal and non-goal-directed movements.

Supplementary Movie 1. Event-related potentials surrounding smartphone touchscreen interactions. Statistics corresponding to one-sample *t*-test corrected for multiple comparisons (MCC, $p < 0.05$).

Supplementary Movie 2. Event-related potentials surrounding touches on a smartphone-like surface.

Supplementary Movie 3. Event-related spectral perturbations surrounding smartphone touchscreen interactions collapsed across the beta-band (12 to 30 Hz) by estimating the 20% trimmed means at each time point. Statistics corresponding to one-sample *t*-test corrected for multiple comparisons, masked T-values are collapsed by using the maximum absolute amplitude).

Supplementary Movie 4. Event-related spectral perturbations surrounding smartphone touchscreen interactions collapsed across the alpha-band (8 to 11 Hz).

Supplementary Movie 5. Event-related potentials surrounding goal-directed movements.

Supplementary Movie 6. Event-related potentials surrounding non-goal-directed movements.

Supplementary Movie 7. Paired-sample *t*-test comparing event-related potentials of goal and non-goal-directed movements. Statistics corrected for multiple comparisons.

Supplementary Movie 8. Event-related spectral perturbations surrounding goal-directed movements collapsed across the alpha-band (8 to 11 Hz).

Supplementary Movie 9. Event-related spectral perturbations surrounding goal-directed movements collapsed across the beta-band (12 to 30 Hz).

Supplementary Movie 10. Event-related spectral perturbations surrounding goal-directed movements collapsed across the gamma-band (31 to 40 Hz).

Supplementary Movie 11. Event-related spectral perturbations surrounding non-goal-directed movements collapsed across the beta-band (12 to 30 Hz).

Supplementary Movie 12. Event-related spectral perturbations surrounding non-goal-directed movements collapsed across the gamma-band (31 to 40 Hz).

Supplementary Movie 13. Event-related spectral perturbations surrounding non-goal-directed movements collapsed across the alpha-band (8 to 11 Hz).

Supplementary Movie 14. Paired-sample *t*-test comparing event-related spectral perturbations for goal and non-goal-directed movements. Statistics corrected for multiple comparisons.

Supplementary Movie 15. Event-related potentials surrounding goal-directed artificial touches.

Supplementary Movie 16. Event-related potentials surrounding non-goal-directed artificial touches.

Supplementary Movie 17. Illustrative video of the experimental setup showing participant scrolling on a smartphone with the movement sensor attached to the right thumb.
