# Supplementary figures and images for: Neural processing of goal and non-goal-directed movements on the smartphone
Source: Neuroimage Rep. 2023 Mar 15;3(2):100164. doi: 10.1016/j.ynirp.2023.100164 (PMC12172746; doi:10.1016/j.ynirp.2023.100164)

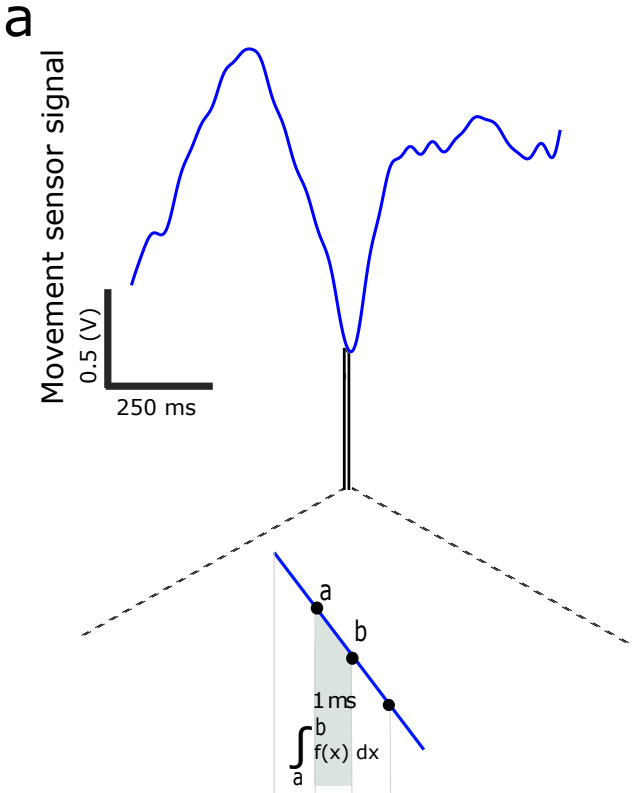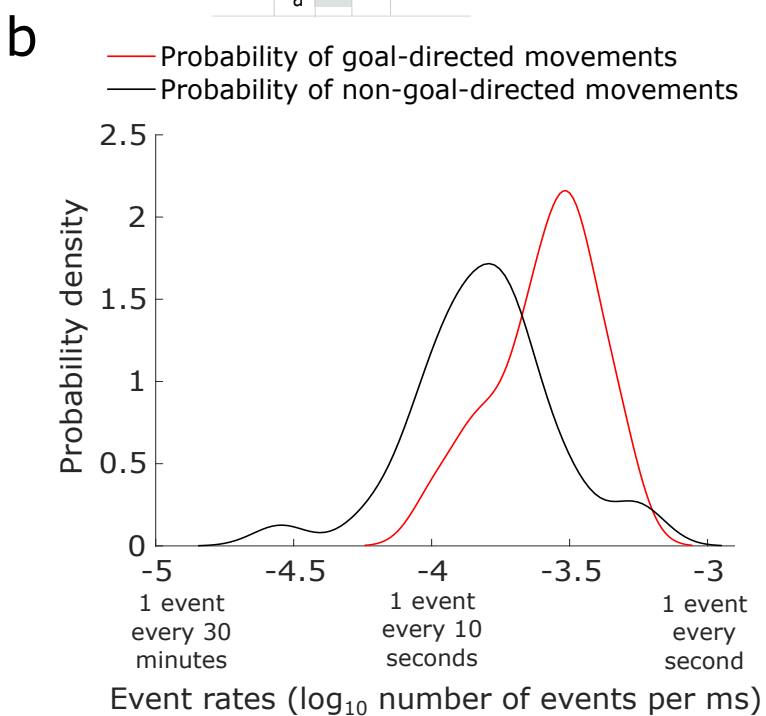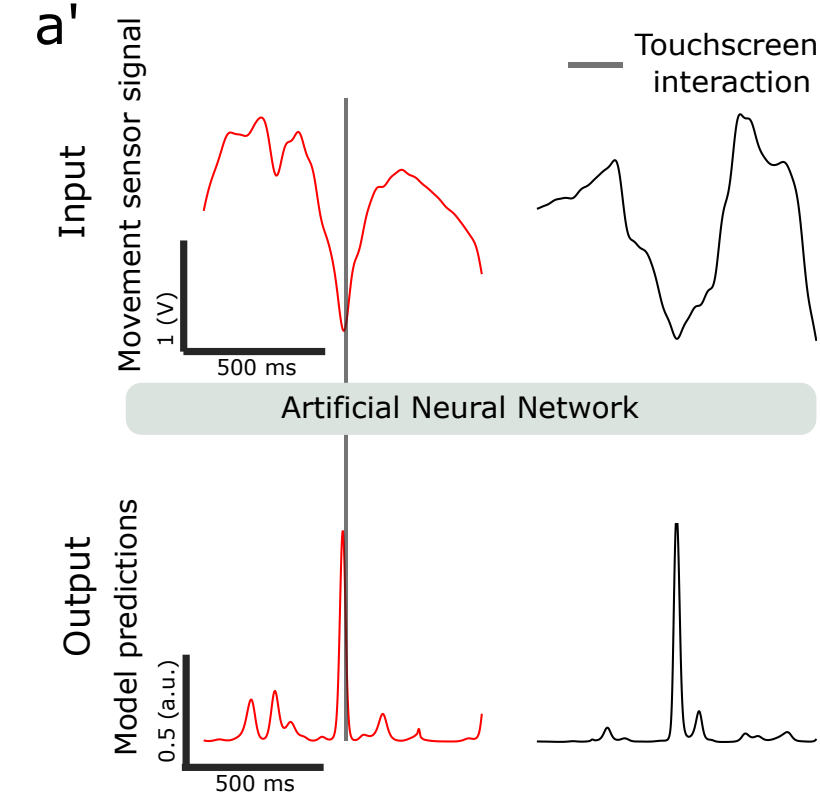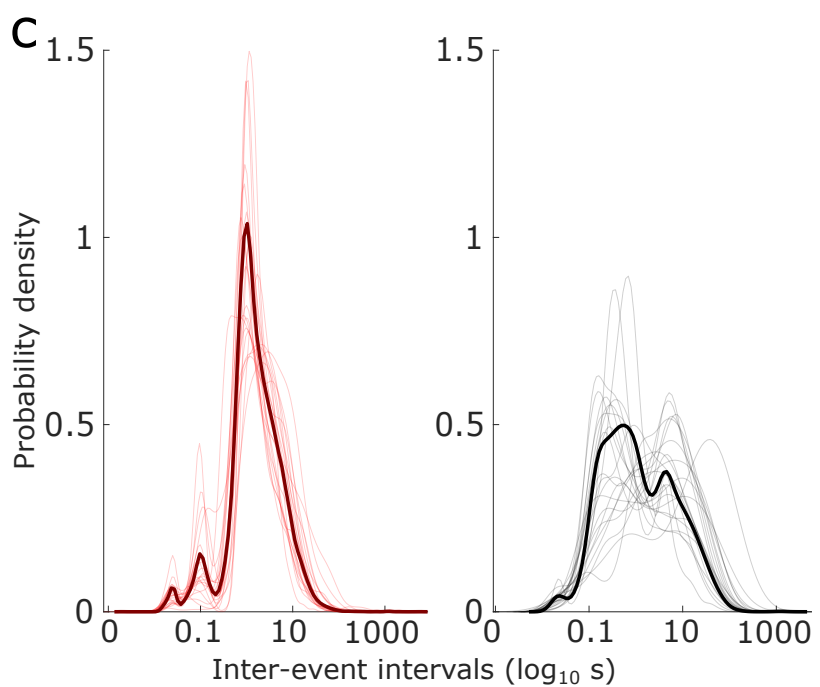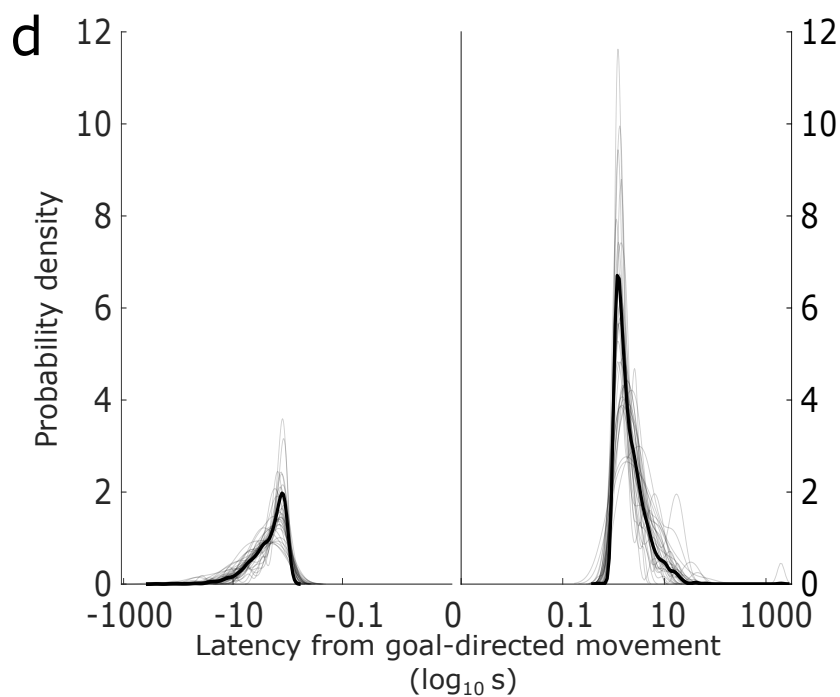

Supplement: Supplementary Figure 1 [file mmc3.pdf]

## a Goal-directed movements

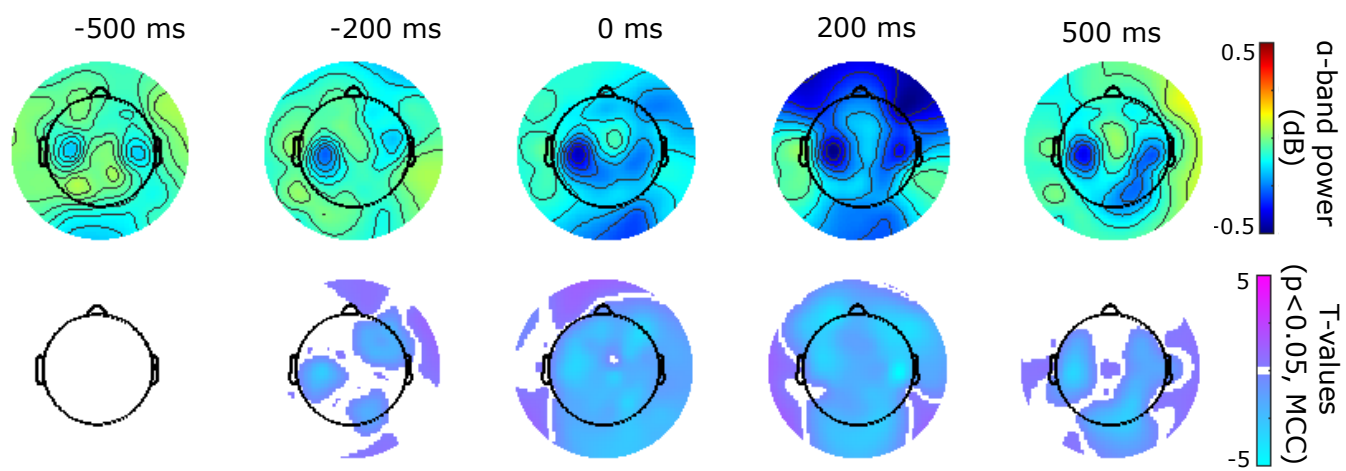

## b Non-goal-directed movements

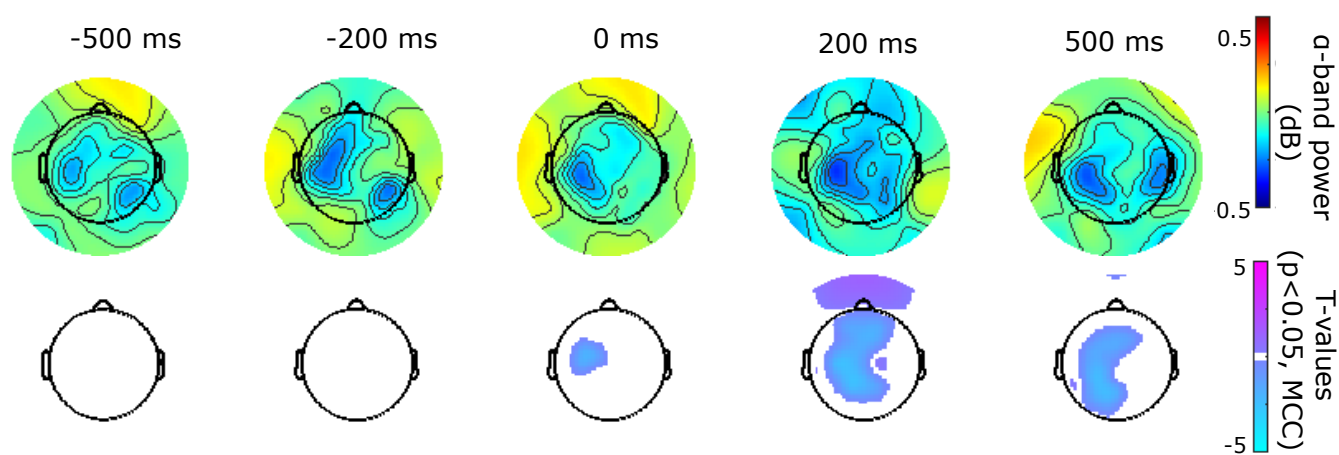

Supplement: Supplementary Figure 4 [file mmc6.pdf]
