# Supplementary material for: Neural processing of goal and non-goal-directed movements on the smartphone
Source: Neuroimage Rep. 2023 Mar 15;3(2):100164. doi: 10.1016/j.ynirp.2023.100164 (PMC12172746; doi:10.1016/j.ynirp.2023.100164)
Supplement: Supplementary Figure 3 [file mmc5.pdf]

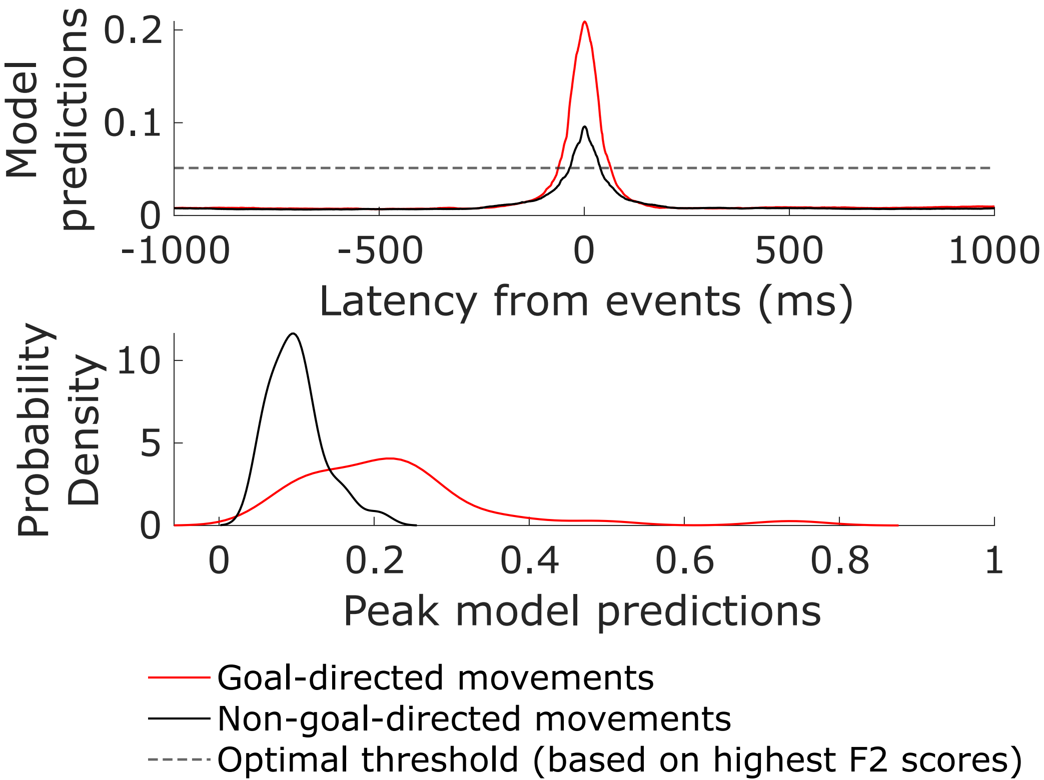

Model predictions
0.2
0.1
0
-1000
-500
0
500
1000
Latency from events (ms)
Probability Density
10
5
0
0
0.2
0.4
0.6
0.8
1
Peak model predictions
Goal-directed movements
Non-goal-directed movements
Optimal threshold (based on highest F2 scores)
