# Supplementary material for: Neural processing of goal and non-goal-directed movements on the smartphone
Source: Neuroimage Rep. 2023 Mar 15;3(2):100164. doi: 10.1016/j.ynirp.2023.100164 (PMC12172746; doi:10.1016/j.ynirp.2023.100164)
Supplement: Supplementary Figure 5 [file mmc7.pdf]

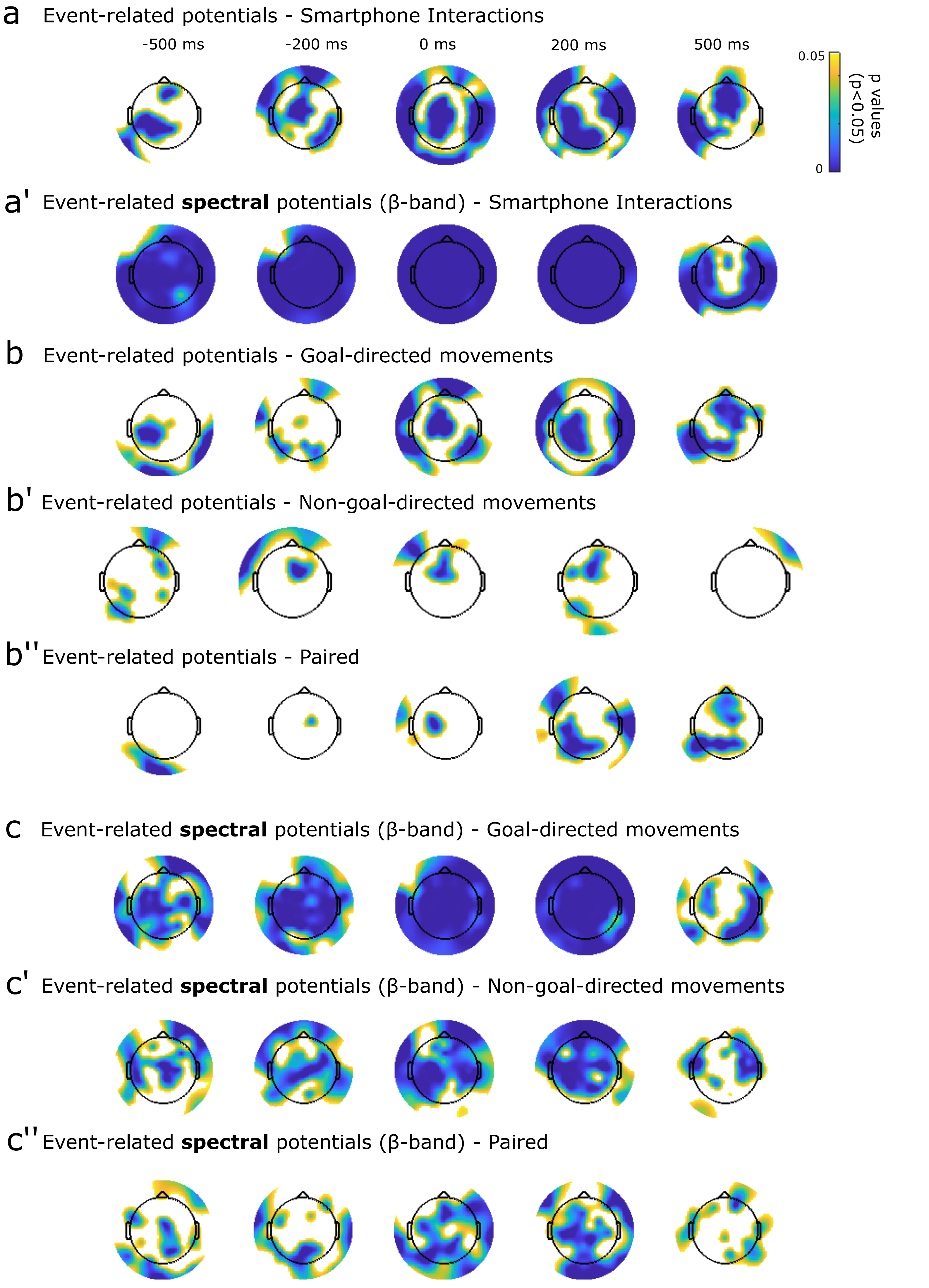
a Event-related potentials - Smartphone Interactions
-500 ms
-200 ms
0 ms
200 ms
500 ms
0.05
0
p values (p<0.05)
a' Event-related spectral potentials (β-band) - Smartphone Interactions
b Event-related potentials - Goal-directed movements
b' Event-related potentials - Non-goal-directed movements
b'' Event-related potentials - Paired
c Event-related spectral potentials (β-band) - Goal-directed movements
c' Event-related spectral potentials (β-band) - Non-goal-directed movements
c'' Event-related spectral potentials (β-band) - Paired
