# Supplementary material for: Neural processing of goal and non-goal-directed movements on the smartphone
Source: Neuroimage Rep. 2023 Mar 15;3(2):100164. doi: 10.1016/j.ynirp.2023.100164 (PMC12172746; doi:10.1016/j.ynirp.2023.100164)
Supplement: Supplementary Table 1 [file mmc9.pdf]

Supplementary Table 1. Descriptive statistics for artificial neural networks trained to identify the goal and non-goal-directed movements

	**Median F2 score**	**Median Precision**	**Median Recall**
Selected participants (N = 32)	0.3403	0.1419	0.5475
All participants (N = 68)	0.3453	0.1490	0.5484
